# Inflammatory Type Focal Cerebral Arteriopathy of the Posterior Circulation in Children: A Comparative Cohort Study

**DOI:** 10.1161/STROKEAHA.123.043562

**Published:** 2024-03-06

**Authors:** Nedelina Slavova, Robin Muenger, Iciar Sanchez-Albisua, Maria Regényi, Gabriela Oesch, Joël Fluss, Annette Hackenberg, Sébastien Lebon, Oliver Maier, Alexandre N. Datta, Sandra Bigi, Sebastian Grunt, Maja Steinlin

**Affiliations:** Support Center for Advanced Neuroimaging, Institute of Diagnostic and Interventional Neuroradiology (N.S.), Inselspital, Bern University Hospital, University of Bern, Switzerland.; Division of Neuropaediatrics, Development and Rehabilitation, Department of Paediatrics (R.M., I.S.-A., M.R., G.O., S.G., M.S.), Inselspital, Bern University Hospital, University of Bern, Switzerland.; Pediatric Radiology, University of Basel Children’s Hospital and University of Basel, Switzerland (N.S.).; Department of Neurology, University Hospital Bern (N.S.), University of Bern, Switzerland.; Institute of Social and Preventive Medicine (S.B.), University of Bern, Switzerland.; Department of Pediatrics, Gynecology and Obstetrics, Pediatric Neurology Unit, University Hospitals of Geneva, Switzerland (J.F.).; Department of Pediatric Neurology, University Children’s Hospital, Zürich, Switzerland (A.H.).; Pediatric Neurology and Neurorehabilitation Unit, Lausanne University Hospital, Switzerland (S.L.).; Division of Child Neurology, Department of Pediatrics, Children’s Hospital, St. Gallen, Switzerland (O.M.).; Department of Pediatric Neurology and Developmental Medicine, University of Basel Children’s Hospital, Switzerland (A.D.).; Division of Pediatric Neurology, Department of Pediatrics, Children’s Hospital Lucerne, Switzerland (S.B.).

**Keywords:** carotid artery, internal, constriction, pathologic, ischemic stroke, posterior cerebral artery, stroke

## Abstract

**BACKGROUND:**

Inflammatory type focal cerebral arteriopathy (FCA-i) in the anterior circulation (AC) is well characterized, and the focal cerebral arteriopathy severity score (FCASS) reflects the severity of the disease. We identified cases of FCA-i in the posterior circulation (PC) and adapted the FCASS to describe these cases.

**METHODS:**

In this comparative cohort study, patients from the Swiss NeuroPaediatric Stroke Registry with ischemic stroke due to FCA-i between January 2000 and December 2018 were analyzed. A comparison between PC and AC cases regarding pediatric National Institutes of Health Stroke Scale score and pediatric stroke outcome measure and FCASS was performed. We estimated infarct size by the modified pediatric Alberta Stroke Program Early Computed Tomography Score in children with AC stroke and the adapted Bernese posterior diffusion–weighted imaging score in the PC.

**RESULTS:**

Thirty-five children with a median age of 6.3 (interquartile range, 2.7–8.2 [95% CI, 0.9–15.6]; 20 male; 57.1%) years with FCA-i were identified. The total incidence rate was 0.15/100 000/year (95% CI, 0.11–0.21). Six had PC-FCA-i. Time to final FCASS was longer in the PC compared with AC; the evolution of FCASS did not differ. Initial pediatric National Institutes of Health Stroke Scale score was higher in children with FCA-i in the PC with a median of 10.0 (interquartile range, 5.75–21.0) compared with 4.5 (interquartile range, 2.0–8.0) in those with AC-FCA-i. Different from the anterior cases, PC infarct volume did not correlate with higher discharge, maximum, or final FCASS scores (Pearson correlation coefficient [r], 0.25, 0.35, and 0.54).

**CONCLUSIONS:**

FCA-i also affects the PC. These cases should be included in future investigations into FCA-i. Although it did not correlate with clinical outcomes in our cohort, the modified FCASS may well serve as a marker for the evolution of the arteriopathy in posterior FCA-i.

Inflammatory type focal cerebral arteriopathy (FCA-i) is an important risk factor for arterial ischemic stroke (AIS) in previously healthy children.^1^

There have been changes in the definition of focal cerebral arteriopathy (FCA) over the years including the inclusion or exclusion of posterior circulation (PC) FCA. After the first description in 1998,^2^ Sébire et al^3^ in 2004 defined a transient cerebral arteriopathy by typical vascular imaging within 3 months after stroke showing unilateral focal or segmental stenosis or occlusion at a typical location, nonprogressing on follow-up imaging 6 months after stroke. Typical locations include the distal part of the internal carotid artery and the initial segments of the anterior, middle, and posterior cerebral arteries (A1, M1, and P1 segments). As a special variant, postvaricella arteriopathy was defined as transient cerebral arteriopathy with a history of acute varicella infection within 12 months before AIS.^4,5^ The term FCA was later defined, and transient cerebral arteriopathy was considered a subset of FCA.^6,7^ The CASCADE (Childhood AIS Standardized Classification and Diagnostic Evaluation) system in 2012 later defined type 2 arteriopathy as unilateral FCA of childhood (FCA), including anterior circulation (AC) with collaterals, AC without collaterals, and PC cases.^8^

In the large international VIPS study (Vascular Effects of Infection in Pediatric Stroke), the definition of FCA was narrowed to unifocal and unilateral stenosis or irregularities of the AC. The VIPS study further subdivided FCA into 3 groups: FCA with intracranial arterial dissection (FCA-d), FCA-i, or FCA undefined cause.^1,9^ FCA-i accounted for the majority of FCA in the VIPS study.^1^ The evolution of FCA-i is characterized by a monophasic natural course, frequently showing early progression (over days to weeks), which reaches a plateau with nonprogression by 6 months. Most individuals improve or remain stable, but complete resolution is seen in only a minority of cases.^2,3^ Herpes simplex virus, enterovirus, mycoplasma pneumonia, or Borrelia burgdorferi infections have been reported as risk factors for FCA-i and AIS.^10–15^

Based on the hypothesis of an inflammatory pathogenesis, corticosteroids are increasingly used to treat FCA-i.^16^ However, there is still no clinical trial data to support this treatment. The planning of forthcoming clinical trials in this field^17^ revealed the need for a quantitative measurement of FCA severity. This led to the development of the FCA severity score (FCASS) based on a subcohort of children with FCA enrolled in the VIPS study.^9,18^ Only children with FCA in AC were included precluding its utility for PC stroke.

Postvaricella arteriopathy affecting the PC has been well described in the literature.^19–21^ A study on postvaricella AIS in Denmark included cases fulfilling clinical and radiological criteria and a positive cerebrospinal fluid test for varicella zoster virus (VZV) desoxyribonucleic acid in combination with intrathecal VZV IgG production. This includes 1 case of postvaricella AIS affecting the PC.^22^ A population-based Swiss study revealed an incidence of PC-AIS of 0.18/100 000 children/year. In 25.6% of these cases, the cause was identified as FCA (FCA-i, FCA-d, and FCA undefined cause).^23^ There is currently no scoring system available to describe these cases in detail.

Children with FCA-i mostly present with hemiparesis or hemiplegia.^22,24–26^ In general, children with AIS in the PC show less specific clinical symptoms and signs than those with AIS in the AC.^23^

The aim of our study was to identify all cases of FCA-i in the Swiss NeuroPaediatric Stroke Registry (SNPSR), adapt the FCASS to encompass PC-FCA-i, and compare clinical and radiological features of FCA-i between PC and AC.

## METHODS

### Data Availability

Anonymized raw data are available and can be shared upon reasonable request.

### Patients

We conducted a retrospective analysis of the nationwide SNPSR, initiated in January 2000, which includes clinical and radiological data on childhood AIS cases in Switzerland.^24^ Hospital-based pediatric neurologists report all cases to the registry in Bern. Follow-up is conducted through questionnaires sent to parents at 2 and 5 years. Short-term follow-up is done by pediatric neurologists. The SNPSR has ethical approval, and consent was obtained from registered patients or their legal guardians.^23^ Details of the SNPSR can be found in previous publications.^24,27^ Some patients from the cohort have also been included in previous studies based on the SNPSR. In one study, it was a Swiss-Australian cohort,^16^ and the other study was children with FCA of inflammatory, dissection, and unknown type.^18^

Childhood AIS was defined as a sudden (focal) neurological deficit with radiological proof of an acute ischemic lesion in the corresponding vascular territory. FCA-i included monophasic FCA, as in postvaricella arteriopathy and transient cerebral arteriopathy.^28^

### Inclusion and Exclusion Criteria

All data of children (aged 1 month to 16 years) with AIS registered between January 2000 and December 2018 with available good-quality neuroimaging at diagnosis and follow-up, with documented abnormalities on vascular neuroimaging, were retrospectively reviewed. In addition, charts from children with a history of varicella infection within the last 12 months before the stroke were also reviewed.

The inclusion criteria were:

1. Childhood stroke due to FCA-i defined by neuroimaging: focal stenosis, irregularity, or banding in any vascular territory. To increase the probability of the inflammatory nature of FCA, the 2 following criteria were added:
  - accompanied by vessel wall contrast enhancement on vessel wall imaging (VWI) or
  - preceded by infection (varicella within 12 months, other infections within a maximum of 8 weeks before stroke) and in the absence of trauma.
2. Monophasic arteriopathy without progression in long-term follow-up.

Exclusion criteria were arterial dissections, moyamoya arteriopathy, and underlying systemic diseases, such as bacterial meningitis, neurofibromatosis, Kawasaki disease, or systemic lupus erythematosus.

### Data Collection

Demographic, clinical, and infectious risk factors, laboratory investigations, and treatment data were extracted from the SNPSR. Incomplete data were supplemented by reviewing the original charts. The pediatric National Institutes of Health Stroke Scale score, obtained from treating physicians or retrospectively performed and neuroimaging analyzed by a trained pediatric neuroradiologist, was collected. Pediatric stroke outcome measure (PSOM)^29^ was gathered from SNPSR or medical charts at discharge, 6 months, and 24 months. The outcome was categorized as either good (total PSOM, 0–0.5) or poor (total PSOM, ≥1). Neuroimaging was retrospectively analyzed by a trained pediatric neuroradiologist (N.S.).

### Radiological Scoring of Arteriopathy Severity

FCASS was applied for assessment of the arteriopathy.^14,16^ To measure the severity of stenosis in the PC, the same principles were applied to the following segments: intracranial vertebral artery, basilar artery (BA), P1 and P2 segments of the posterior cerebral artery, the superior cerebellar artery, the anterior inferior cerebellar artery, and the posterior inferior cerebellar artery (Table S1). We first applied FCASS to the PC as no standardized posterior FCASS exists.

FCASS was evaluated using magnetic resonance angiography or, if unavailable, computed tomography angiography. For analysis, we considered baseline FCASS, maximum FCASS during follow-up (if multiple measurements were available), and final FCASS (if follow-up imaging was conducted >6 months after the stroke).

### Radiological Scoring of Infarct Size

Infarct size on admission for AC stroke was calculated using the modified pediatric Alberta Stroke Program Early Computed Tomography Score,^30–32^ with a maximum of 30 points (15 for each side). Because the pediatric Alberta Stroke Program Early Computed Tomography Score does not cover most locations in the PC, the modified Bernese diffusion–weighted imaging score was used for stroke in PC,^33^ adding up to a maximum of 22 points (for details, see Tables S2 and S3).

### Statistical Analyses

First, variables were analyzed descriptively. The incidence rate of FCA-i in the PC in Swiss children was calculated using data from the Swiss Federal Statistical Office. Comparisons were made between groups with strictly anterior or posterior involvement. Patients with missing data were excluded. Nonparametric statistics (the Wilcoxon rank-sum test and the signed-rank test) were used for between-group comparisons, the Fisher exact test for binary outcomes, and the Spearman correlation coefficient for subgroup correlations. A significance level of *P*<0.05 was applied. R statistical software (version 3.6.1) was used for analysis. The STROBE cohort checklist was followed in report writing.^34^

## RESULTS

### Study Population and Epidemiology

During the study period (January 2000 to December 2018), 301 children with AIS (aged 1 month to 16 years) were registered in the SNPSR. Among them, 126 cases with either documented vascular abnormalities or recent acute VZV infection were clinically reviewed. After excluding cases with alternative diagnoses, central adjudication of images was performed. Ninety-one children were excluded: 9 had intracranial dissection (FCA-d), 11 were classified as FCA undefined cause, 10 had extracranial dissection, 1 had internal carotid artery occlusion, and 11 had arteriopathy due to other pathologies. Additionally, 8 children had cardioembolic stroke and 9 had progressive central nervous system angiitis. In 32 cases, no arteriopathy was found during radiological review.

FCA-i was found in 35 children of which 5 cases fulfilled all FCA-i inclusion criteria but interestingly affected AC and PC simultaneously. Twenty-three cases were included because of vascular abnormalities recorded in the SNPSR (PC, 4; AC, 16; and both, 3) and 12 because of a history of varicella infection (PC, 2; AC, 8; and both, 2).

Vessel wall enhancement was present in 6 of 8 cases where VWI was performed. Eighteen AC, 5 PC, and 5 cases in the third group showed the typical course of FCA-i; 19 cases had evidence of clinically relevant infection.

The overall incidence rate of FCA-i in our Swiss cohort was 0.15/100 000 children/year (95% CI, 0.11–0.21). AC was seen in 24 children and PC in 6 with an incidence rate of 0.101 (95% CI, 0.067–0.150) and 0.025 (95% CI, 0.01–0.06) respectively.

Table 1 displays the baseline characteristics of all patients; cases affecting both circulations simultaneously were not included in statistical analyses. Significant differences between AC and PC were found in the initial presentation: patients with AIS affecting the AC presented more often with facial palsy than those with PC stroke. The initial pediatric National Institutes of Health Stroke Scale score was significantly higher in the PC group, and they reported more often headache as a leading symptom. Tetra paresis was significantly more often found among cases with posterior FCA-i. Intravenous lysis was performed more often if the PC was affected. Fifteen children received steroid treatment (10 AC [42%] and 1 PC [29%], and 4 in the group where both circulations were affected).

**Table 1.**
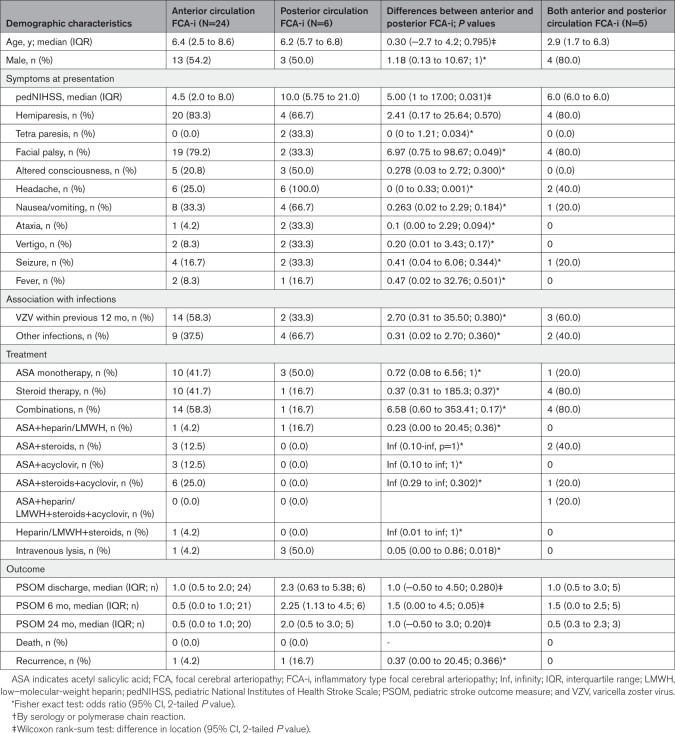
Summarized Baseline Characteristics of the Patients

Details on baseline characteristics, radiological findings, treatment, and outcome of PC cases and those with both circulations affected are summarized in Tables S4 and S5.

Fifteen patients were tested specifically for VZV infection either in blood or cerebrospinal fluid samples; in 7 patients (1 in PC, 3 in AC, and 3 in the group where both circulations were affected) VZV was found by a polymerase chain reaction test in the cerebrospinal fluid. Further details on VZV testing are summarized in Table S6.

### FCASS

**Figure 1.**
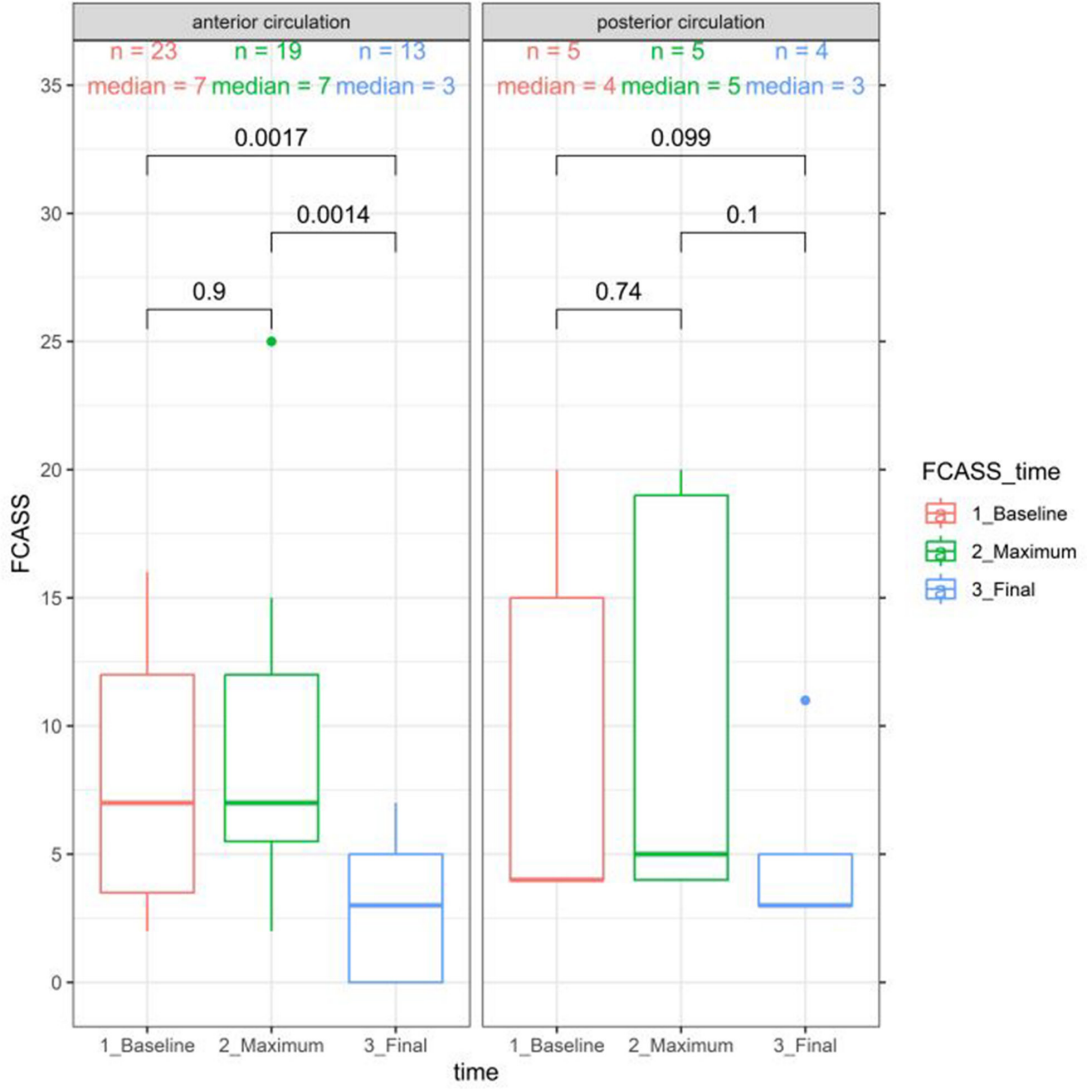
**Baseline maximum and final focal cerebral arteriopathy severity score (FCAS).** Comparison by the Wilcoxon rank-sum test and signed-rank test; 2-sided *P* value indicated.

Table 2 summarizes FCASS results for the anterior and posterior groups. Baseline FCASS was obtained mainly through magnetic resonance angiography, except for a few cases that used conventional angiography or computed tomography angiography (1 PC and 3 AC). Follow-up FCASS was consistently obtained via magnetic resonance angiography. The neuroimaging follow-up duration differed significantly between the 2 groups, with a longer median time for the posterior group (300 weeks) compared with the anterior group (64 weeks; *P*=0.043). No significant differences were found in scores or other time periods between the groups. Figure 1 illustrates the FCASS evolution in the AC and PC. Improvement was observed after 6 months, with median improvements of 4 points in the anterior group and 5.5 points in the posterior group. Initial worsening appeared more pronounced in cases involving the PC, but there was no significant difference between the groups. No correlation was found between the pediatric National Institutes of Health Stroke Scale score or PSOM and FCASS for either group, and no correlation was evident between FCASS and infarct size. Direct comparison of infarct volume between the anterior and posterior cases was not possible due to the different scoring systems used.

**Table 2.**
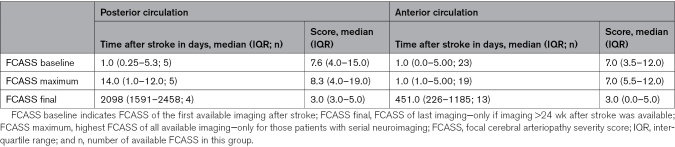
FCASS Summary at Different Time Points

## DISCUSSION

The aim of this study was to characterize inflammatory FCA cases in the PC registered in the SNPSR and compare them to anterior FCA-i cases. In this cohort, we observed FCA confined to the PC in one-fifth of the cases. This is consistent with existing data on PC-AIS in childhood.^19–23^ Understanding the clinical presentation and implementing a severity scoring system for arteriopathy are crucial.^35^ Comparison of PC and AC cases showed a similar age distribution, with a male predominance that is consistent with childhood AIS in general.^24^ As previously reported,^23,36,37^ PC-FCA-i cases often presented with nonspecific symptoms, particularly headache, and had higher pediatric National Institutes of Health Stroke Scale scores at presentation, which might be because of brainstem involvement. Also consistent with the literature, facial palsy was more common in the anterior group^37^; 60% of AC and 33% of PC cases were associated with a VZV infection. Although this difference was not significant, the small sample was clearly underpowered to detect such an association with a high probability. All these between-group comparisons must be interpreted cautiously. Concerning VZV infection, because the decision to test for VZV in the first place was purely clinical, we cannot distinguish whether there really is no association, or whether it is a sampling bias of our cohort.

**Figure 2.**
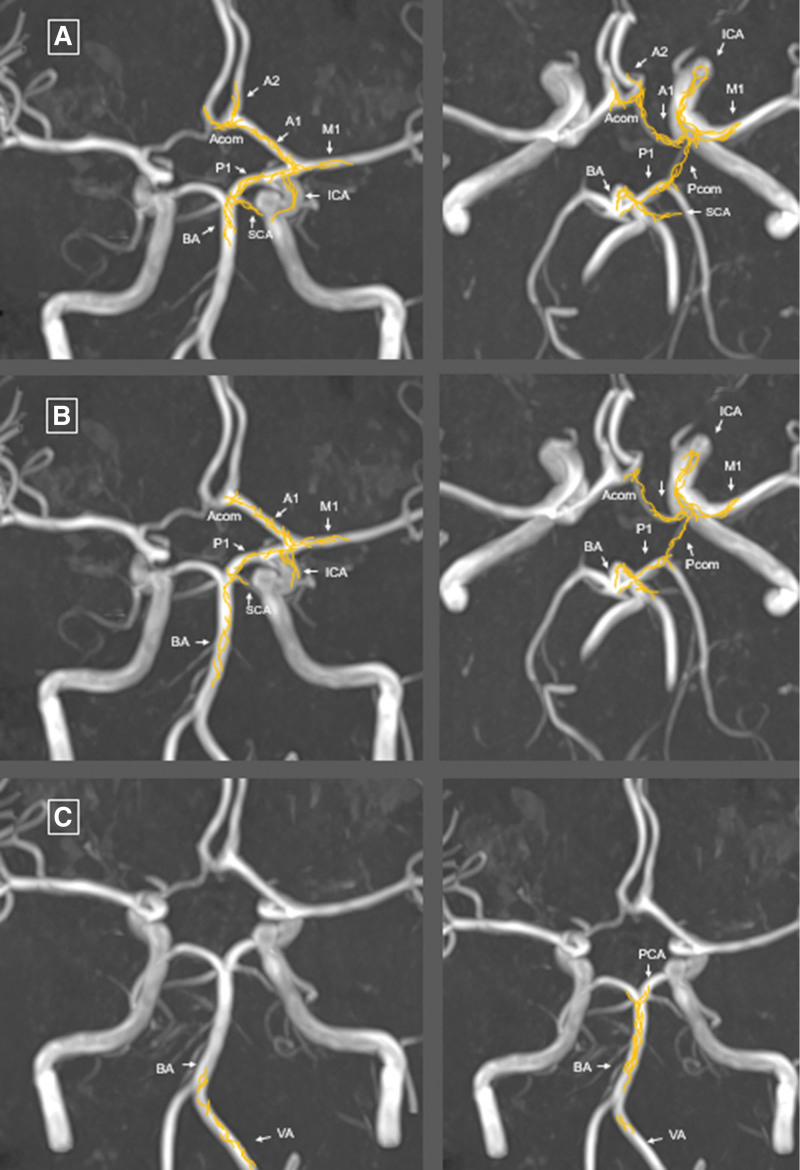
**Illustration depicting the distribution of nerve fibers superimposed on magnetic resonance images, as described by Arbab et al.^40^** Applied from Arbab et al^40^ using neuroanatomical tracing techniques after tracer injection into (**A**) the trigeminal ganglion, (**B**) the superior cervical ganglion, and (**C**) first (left image) and second (right image) spinal ganglia. The distribution of the anterogradely labeled nerve fibers (in yellow) was mapped on time-of-flight magnetic resonance angiography and maximum intensity projection reconstruction imaging of the cerebral blood vessels. Acom indicates anterior communicating artery; BA, basilar artery; ICA, internal carotid artery; PCA, posterior cerebral artery; Pcom, posterior communicating artery; SCA, superior cerebellar artery; and VA, vertebral artery.

The reason why arteriopathy occurs in the proximal segments of the circle of Willis remains unclear. It has been hypothesized that when VZV reactivates in ganglia,^38^ the viral capsid may migrate by axonal retrograde transport (sensory nerve fibers) to the vessel wall of these vessels and replicate and induce a local inflammatory response after transmural spread. This is supported by a report that showed VZV in the vessel wall of the MCA in a child who died of a stroke.^5^ Experimental studies in monkeys have shown that intracerebral arteries receive afferent fibers from trigeminal and superior cervical ganglia (harboring latent varicella infection), not only to the carotid T-junction but to a lesser extent also to the posterior, rostral BA, posterior cerebral artery, and superior cerebellar artery.^39^ A study in rats showed that fibers from the first and second spinal ganglion reach intracranial portions of the ipsilateral vertebral artery and the BA (as represented in Figure 2).^40^ This may explain the distribution of FCA-i in both the AC and PC.

A characteristic feature of FCA-i is the monophasic course of the disease without progression on long-term follow-up. At initial presentation, it is difficult to distinguish between ongoing vasculitis and FCA-i, especially in the presence of multiple lesions. In this retrospective analysis, we were able to include those cases with multiple lesions that showed the typical evolution and could be retrospectively classified as FCA-i. Considering the hypothetical pathophysiology mentioned above, we can even expect reactivation to occur simultaneously in different vessels in the described territory.

Interestingly, we had children who had FCA-i in the AC and PC at the same time according to imaging and clinical course. However, we must admit that 2 of them had an underlying problem, which is not usually associated with FCA-i. In 3 of them, positive VZV polymerase chain reaction indicates a past VZV infection. We cannot confirm that FCA-i can occur in both circulations at the same time. This possibility should be kept in mind, and we hope that future research will provide the answer.

A major challenge is the distinction between FCA-d and FCA-i.^23^ In the early years of the registry, FCA-i was not defined or scored. With the goal of not to miss any case, which, in retrospect, would be classified as FCA-i, initial inclusion criteria were taken broad. This explains why so many cases had to be excluded upon clinical and radiological review.

All FCA cases were reviewed for the typical signs of dissections such as ectasia, fusiform aneurysm, intimal flap, intramural hematoma, or pseudoaneurysm. Consistent with the literature, a history of head trauma was considered to support the diagnosis of FCA-d.^1^ The presence of multiple vessel involvements (as typical for PC cases) makes the diagnosis of a dissection unlikely. Additionally, all lesions in our cases are localized in the typical region of the wandering of the virus along sympathetic and sensory fibers such as P1, the basilar artery, and segments 4 and 5 of the vertebral arteries. In the cases affecting the BA, the length of the lesion present is atypical for an intracranial dissection. Considering all these points, we are convinced that our 6 cases in the PC are in fact FCA-i and with a high degree of confidence, not FCA-d.

It is difficult to make the diagnosis of FCA-i in the acute phase. Most specialists agree that a definite diagnosis can only be made by observing the course of the disease. VWI might be a helpful diagnostic tool in this early phase but is still not considered to be a diagnostic criterion. There is only limited data available on VWI in the acute phase because the use of intravenous contrast media must be justified in each case.

Regarding treatment, intravenous thrombolysis was more common when the PC was affected, particularly if the BA was involved, reflecting the severity of the acute disease. However, due to the lack of randomized controlled trials, treatment recommendations for recanalization therapy in children with AIS are primarily based on expert opinion.^41^ In the context of FCA-i treatment, we suggest using mechanical thrombectomy cautiously and only in selected patients with major vessel occlusion (eg, carotid T or M1). Intervention in vessels with underlying inflammation may lead to unsuccessful recanalization or complications. Therefore, intravenous or intra-arterial thrombolysis (distant from the local lesion) should be preferred over thrombectomy. Median PSOM at 6 and 12 months was higher in the posterior group (Table 1), but the difference did not reach statistical significance, likely due to the study’s limited power. Both AC-FCA-i and PC-FCA-i cases had a median PSOM of 1, consistent with the original VIPS study.^9^

To assess arteriopathy in the PC, we adapted the FCASS scoring system for PC-FCA. However, validation of the posterior FCASS was limited by the small number of cases, highlighting the need for a larger patient cohort for this purpose.

We found no correlation between infarct size and FCASS or between PSOM and FCASS in cases involving the PC. Part of the explanation may be the different association between infarct size and clinical outcome in PC due to anatomic features and different dimensions. Furthermore, the small sample size may well have prevented the detection of possible correlations.

The most important limitation of our study is the small sample size, which makes comparison and correlation difficult. Even over this large time span, only a few cases of FCA-i within the PC were registered in Switzerland.

Because of the small sample size, levels of significance of between-group comparisons must be interpreted with caution. However, we still consider our sample representative. Given the acquisition process and the well-established status of the SNPSR, we are convinced that most cases of childhood AIS during this time would have been captured. Thus, the conclusions drawn from our study still provide a solid basis for clinical decisions, highlighting the need for prospective randomized trials, especially regarding treatment regimens.

During the whole study period, VZV vaccination was not part of the national immunization plan in Switzerland, and the vaccination status of the children was not recorded. According to the Intercontinental Medical Statistics (IQVIA), MMRV vaccine market data for Switzerland in 2018, VZV uptake in Swiss children was ≈10%.^42^ The consequential high prevalence of wild-type VZV in our population may limit the generalizability of our results to a more fully immunized population.

Another limitation is the lack of standardized guidelines for follow-up imaging. Because this is a retrospective analysis of a population-based registry and the patients were recruited over a long period of time, the availability of imaging studies and the imaging protocols have changed and differ between cases. Vessel wall enhancement can be present in FCA-i and was present in 6 of 8 patients where VWI was performed.^43^

## CONCLUSIONS

We conclude that FCA-i may be found in the PC. Thus, further studies in this field should consider including cases of FCA-i in PC. Children with posterior FCA-i present more often with nonspecific symptoms. The modified FCASS may well serve as a marker for the evolution of disease but needs external validation.

## ARTICLE INFORMATION

### Acknowledgments

The authors thank all participants of the Swiss NeuroPaediatric Stroke Registry and their parents. The authors also thank Heather J. Fullerton (University of California San Francisco) for her valuable input in the discussion, Charles Grose from the University of Iowa Hospital, Iowa City, for pointing our interest to the explanations of localizations of inflammatory type focal cerebral arteriopathy, and all the coworkers of the Swiss NeuroPaediatric Stroke Registry.

### Sources of Funding

This study was supported by the Swiss Heart Foundation.

### Disclosures

None.

### Supplemental Material

STROBE Checklist

Figure S1

Tables S1–S3

## Supplementary Material


