# Supplementary material for: Inflammatory Type Focal Cerebral Arteriopathy of the Posterior Circulation in Children: A Comparative Cohort Study
Source: Stroke. 2024 Mar 6;55(4):1006–14. doi: 10.1161/STROKEAHA.123.043562 (PMC10962439; doi:10.1161/STROKEAHA.123.043562)
Supplement: Supplementary file 1 [file str-55-1006-s001.pdf]

Inflammatory Type Focal Cerebral Arteriopathy of the Posterior Circulation in Children:

a comparative cohort study

Supplemental Material

Supplemental Table S1. Extended Focal Cerebral Arteriopathy Severity Score (FCASS) for the anterior and posterior circulation

Supraclinoidal ICA	MCA		ACA		VA	BA	PCA		SCA	AICA	PICA	Delta Bonus	Total points
	M1	M2	A1	A2			P1	P2					

Legend: Points allocation: 0=no involvement; 1=irregularity or banding with no stenosis; 2 =stenosis<50%; 3=stenosis >50%; 4=occlusion. Delta Bonus: +1 if interval worsening not captured; -1 if interval improvement is not captured by the sum score. BA, vertebral artery; each segment is assigned a graded severity score: 0, no involvement; 1, irregularity or banding with no stenosis; 2, stenosis <50% reduction in diameter; 3, stenosis >50% reduction in diameter; and 4, occlusion. Due to the small size of A2 and M2 branches as well as the SCA (superior cerebellar artery), PICA (posterior inferior cerebellar artery) and AICA (anterior inferior cerebellar artery), any stenosis of those segments is scored as 3 (with no option to score as 2); hence, these segments are classified into 1 of only 4 categories (scored 0, 1, 3, or 4).

Supplemental Table S2. Modified Bernese Posterior Stroke Score

Lesion location	Uni-/bilateral	Pyramidal tract affected
Medulla		
Pons		
Mesencephalon		
Cerebellum<1/3		N/A
Cerebellum>1/3		N/A
Thalamus		N/A
Temporo-occipital		N/A
Parietal		N/A

Legend: Unilateral lesions are assigned 1 point, bilateral lesions 2 points; points for lesions involving the pyramidal tract are doubled because of their greater impact on the clinical status. One point is assigned for infarcts involving less than one third of a cerebellar hemisphere, and 2 points for involvement of more than one third. N/A=not applicable. The total score is the sum of all locations.

Supplemental Table S3. Modified pediatric Alberta Stroke Program Early Computed Tomography Score (pedASPECTS)

	MCA							ACA		PCA		Subcortical				Sum
	M1	M2	M3	M4	M5	M6	Ins	A1	A2	P1	P2	Caud	Lent N	IC	Thal	
L																
R																

Legend: PedASPECTS score includes supratentorial regions of the anterior, middle and posterior cerebral artery territories in each hemisphere as well as subcortical regions supplied by different arteries. One point is assigned to each region affected by ischemia; up to a total pedASPECTS of 30 for maximal severity (15 per hemisphere). MCA=middle cerebral artery; ACA=anterior cerebral artery; Caud= caudate nucleus; LentN=lentiform nucelus; IC=internal capsule; Thal=thalamus; L=left; R=right.

Supplemental Table S4: Detailed results of cases in the posterior circulation

**Cases with exclusive involvement of posterior circulation**											
**ID**	**Sex/ age**	**Acute main clinical symptoms**	**Initial neurological examination**	**ped NIH SS**	**Infection/ Inflamma-tion**	**Imag-ing modal-ity**	**Vessel s**	**Anatomical location of stroke**	**Treatment**	**Course of FCA-i**	**PSOM**
2	F/5y	L transient sensory loss in upper and lower extremity, weakness in lower extrem-ity	initially normal;<br>L hemiparesis after 2 days	5	streptococc al angina	MRA, angi-ography, US	PCA	R thalamus	ASA;<br>heparin i.v. (15d);<br>phenpro-coumon p.o.	improvement	Discharge: 0<br>6M: 0<br>24M: 0
3	M/7y	R progressive sensory and motor deficit	R hemiparesis;<br>L acute otitis media	5	fever and otitis media	MRA	PCA	L thalamus	ASA	improvement	Discharge: 1<br>6M: 1<br>24M: NA
13	M/10y	headache, subfebrile temperature, cough, rhinitis, vomiting, focal seizure	R hemiparesis, hypertonia and hyperreflexia; truncal hypotonia, aphasia, apraxia	12	viral upper respiratory tract infection	MRA	PCA, VA, BA	R cerebellum<br>L pons<br>L thalamus<br>L temporal lobe	ASA;<br>i.v. lysis	initial worsening, then improvement	Discharge: 3.5<br>6M: 3<br>24M: 2
15	M/6y	vertigo, loss of muscle tone, headache,	uncoordinate d eye movements, ataxia,	25	abdominal pain and vomiting	CTA and MRA	BA	pons<br>L cerebellum	ASA;<br>i.v. lysis;<br>LMWH;	improvement	Discharge: 6<br>6M: 5<br>24M: 3

		abdominal pain, vomiting	hyperactive MTR both sides, pos. Babinski sign bilaterally						phenobarbital		
22	F/7y	headache, nausea, vomiting, loss of equilibrium, speech problems	R hemiparesis R facial palsy L ataxia	8	VZV infection 1 month prior to stroke; blood serology IgG and IgM positive	MRA	PCA, BA, VA	pons L cerebellum	ASA	improvement	Discharge: 0.5 6M: 1.5 24M: 0.5
26	F/6y	headache, nausea, vertigo	LOC, deviation of gaze to the L	24	VZV: PCR and serology positive in liquor	MRA	BA, VA	pons R cerebellum R thalamus	i.v. lysis; heparin; amoxicillin/clavulanate 8d) morphine (6d); acyclovir (11d); methylprednisolone (4d); phenprocoumon (6M)	improvement	Discharge: 6.5 6M: 6 24M: 5

Legend: M = male; F = female; R = right side; L = left side; MTR = muscle tendon reflexes; LOC = loss of consciousness; VZV = varicella zoster virus; PCR = polymerase chain reaction; MRA = magnetic resonance angiography; CTA = computer tomography angiography; US = neurovascular ultrasonography; DB = dark blood imaging; ICA = supraclinoid internal cerebral artery; ACA = anterior cerebral artery; MCA = middle cerebral artery; PCA = posterior cerebral artery; BA = basilar artery; VA = intracranial vertebral artery; ASA = acetylsalicylic acid; d = days; w = weeks; M = months; PSOM = pediatric stroke outcome measure; NA = not available

Supplemental Table S5: Detailed results of cases affecting both circulations

Cases in whom both anterior and posterior circulation are affected											
ID	Sex/Age	Acute main clinical symptoms	Initial neurological examination	ped NIH SS	Infection/ Inflammation	Imaging modality	Vessels	Stroke localisation	Treatment	Course of FCA-i	PSOM
31	M/6y	headache, R motor deficit, facial asymmetry, speech problems	R hemiparesis, R facial palsy, aphasia, deviation of gaze to L	9	streptococcal angina	MRA, US	ICA, MCA, PCA	L internal capsule, L temporal cortex L basalganglia	ASA	improvement	Discharge: 4 6M: 1.5 24M: 0.5
32	F/12y	headache, L motor and sensory deficit	L hemiparesis, L facial palsy	6	Hashimoto-thyroiditis	MRA, US	ICA, MCA, ACA, PCA	R internal capsule, R thalamus	ASA; prednisolone (5d), subsequent tapering (2M)	improvement	Discharge: 0 6M: 0 24M: 0
33	M/2y	R facial asymmetry, R motor deficit	R facial palsy	6	VZV: liquor PCR positive; oligoclonal bands	MRA (+ DB), US	ICA, MCA, ACA, PCA	L basal ganglia	ASA; acyclovir (14d); methylprednisolone (4d), subsequent tapering	improvement	Discharge: 0.5 6M: 0 24M: NA
34	M/3y	L motor deficit	L hemiparesis, L hypertonia (upper extremity)	2	VZV: liquor PCR positive	MRA, US	ICA, MCA, PCA	R basal ganglia	ASA; prednisolone (5d), subsequent tapering	initial worsening, then improvement	Discharge: 1 6M: 2.5 24M: NA

35	M/2y	R motor deficit, vomiting	L facial palsy, L hemiparesis, R focal tonic clonic seizure	6	VZV: liquor PCR positive	MRA	MCA, ACA, PCA, BA	R basal ganglia, R thalamus R occipital lobe, R cerebellum, L parietal lobe	ASA, acyclovir, LMWH, prednisolone (6d), subsequent tapering	initial worsening, then improvement	Discharge: 3<br>6M: 6<br>24M: 4

Legend M = male; F = female; R = right side; L = left side; MTR = muscle tendon reflexes; LOC = loss of consciousness; VZV = varicella zoster virus; PCR = polymerase chain reaction; MRA = magnetic resonance angiography; CTA = computer tomography angiography; US = neurovascular ultrasonography; DB = dark blood imaging; ICA = supraclinoid internal cerebral artery; ACA = anterior cerebral artery; MCA = middle cerebral artery; PCA = posterior cerebral artery; BA = basilar artery; VA = intracranial vertebral artery; ASA = acetylsalicylic acid; d = days; w = weeks; M = months; PSOM = pediatric stroke outcome measure; NA = not available

Supplemental Table S6- VZV infections

	Total	Posterior circulation	Anterior circulation
	(N=30)	(N=6)	(N=24)
anamnestic VZV within 12 months			
No	14 (46.7%)	4 (66.7%)	10 (41.7%)
Yes	16 (53.3%)	2 (33.3%)	14 (58.3%)
VZV tested			
Yes*	11 (36.7%)	2 (33.3%)	9 (37.5%)
No	19 (63.3%)	4 (66.7%)	15 (62.5%)
VZV-IgA in CSF			
positive	0 (0%)	0 (0%)	0 (0%)
negative	1 (100%)	0 (0%)	1 (100%)
not tested	29	6	23
VZV-IgM in CSF			
positive	0 (0%)	0 (0%)	0 (0%)
negative	2 (100%)	0 (0%)	2 (100%)
not tested	28	6 (100%)	22
VZV-IgG in CSF			
positive	3 (60%)	0 (0%)	3 (60%)
negative	2 (40%)	0 (0%)	2 (40%)
not tested	25	6 (100%)	19
VZV PCR in CSF			
positive	4 (50%)	1 (50%)	3 (50%)
negative	4 (50%)	1 (50%)	3 (50%)
not tested	22	4	18
VZV-IgM in blood			
positive	1 (14.3%)	1 (50%)	0 (0%)
negative	6 (85.7%)	1 (50%)	5 (100%)
not tested	23	4	19
VZV-IgG in blood			
positive	5 (62.5%)	2 (100%)	3 (50%)
negative	3 (37.5%)	0 (0%)	3 (50%)
not tested	22	4	18

Legend: CSF = Cerebrospinal Fluid; IgA = Immunoglobulin A; IgM = Immunoglobulin M; PCR = Polymerase Chain Reaction; VZV = Varicella Zoster Virus.

* in 3 patients (all in the AC group) both IgG and PCR, in 2 both IgG and PCR was positive and in one, both was negative.

IgG ratios between CSF and blood are not available from the SNPSR.

Supplemental Figure S1. Example of acute imaging in strictly posterior involvement

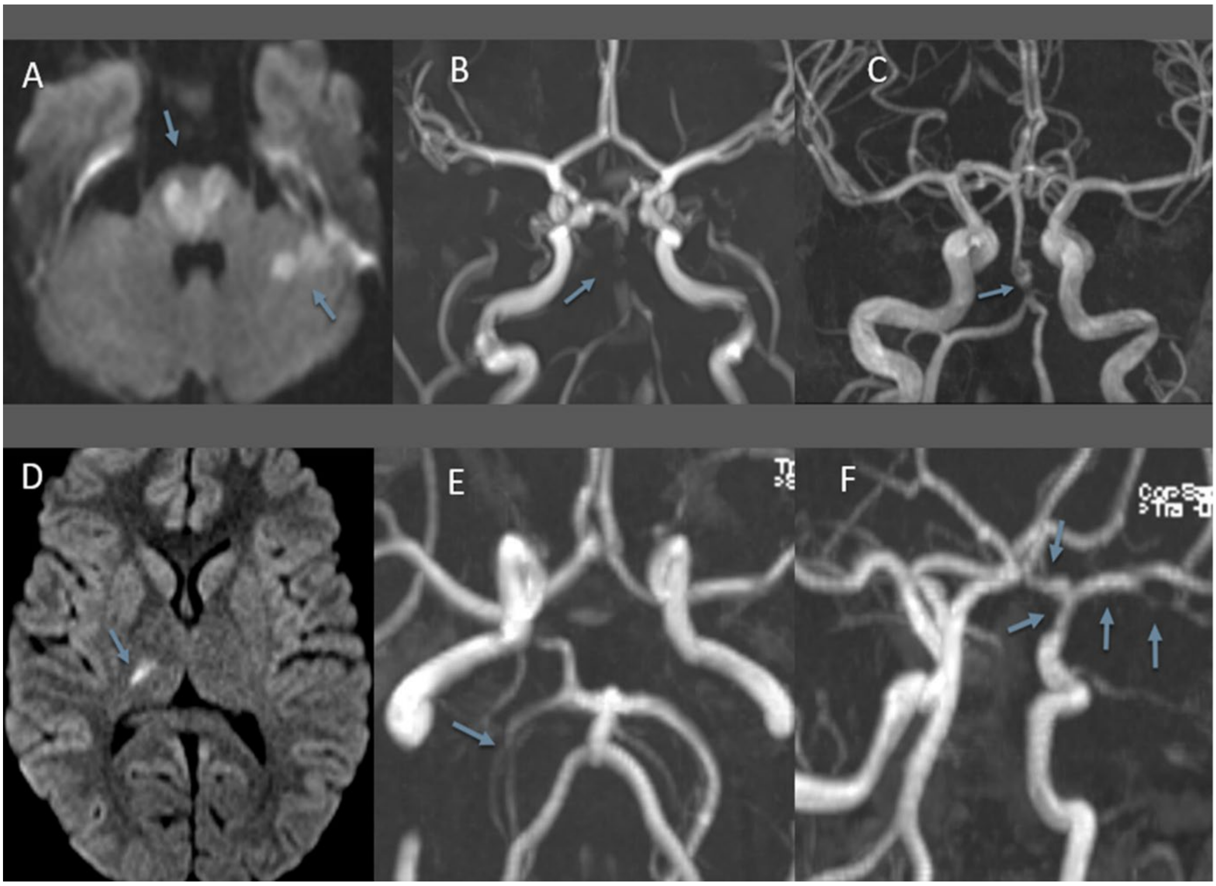

Legend: Upper row: example of acute imaging from a child with strictly posterior involvement. Images from a 5-year-old boy with posterior circulation stroke and FCA-i. (A) Diffusion-weighted imaging (DWI) with a modified Bernese score of 4 points (bilateral involvement of pons; <1/3 of left cerebellar surface). Time-of-flight (ToF) magnetic resonance angiography (MRA) maximum intensity projection (MIP) reconstruction imaging of the cerebral blood vessels at presentation (B) shows occlusion affecting the basilar artery with a FCASS of 4. Follow-up imaging 7 years later (C) shows an improvement with residual high-grade basilar artery stenosis (>50%, FCASS=3). Lower row: images from a 12-year-old girl with FCA-i involving both the anterior and posterior circulation. (D) DWI revealed right thalamic infarction (DWI score of 1 point). ToF-MRA MIP images (E) and (F) show multiple vessel abnormalities (FCASS=13): distal stenosis of the P2 segment of the posterior cerebral artery on the right, stenosis at the carotid-T, A1 and A2 segment of the anterior cerebral artery on the left; banding of M1 segment of the left middle cerebral artery and stenosis of the proximal left M2 segment (arrows).
